# A Novel Hydroxamate-Based Compound WMJ-J-09 Causes Head and Neck Squamous Cell Carcinoma Cell Death via LKB1-AMPK-p38MAPK-p63-Survivin Cascade

**DOI:** 10.3389/fphar.2018.00167

**Published:** 2018-03-01

**Authors:** Chia-Sheng Yen, Cheuk-Sing Choy, Wei-Jan Huang, Shiu-Wen Huang, Pin-Ye Lai, Meng-Chieh Yu, Ching Shiue, Ya-Fen Hsu, Ming-Jen Hsu

**Affiliations:** ^1^Department of General Surgery, Chi Mei Medical Center, Tainan, Taiwan; ^2^Department of Emergency, Min-Sheng General Hospital, Taoyuan, Taiwan; ^3^Department of Community Medicine, En Chu Kong Hospital, New Taipei, Taiwan; ^4^Graduate Institute of Pharmacognosy, Taipei Medical University, Taipei, Taiwan; ^5^Department of Medical Research, Taipei Medical University Hospital, Taipei, Taiwan; ^6^Graduate Institute of Medical Sciences, College of Medicine, Taipei Medical University, Taipei, Taiwan; ^7^Department of Pharmacology, School of Medicine, College of Medicine, Taipei Medical University, Taipei, Taiwan; ^8^Division of General Surgery, Department of Surgery, Landseed Hospital, Taoyuan, Taiwan

**Keywords:** aliphatic hydroxamate, liver kinase B1 (LKB1), p63, survivin, head and neck squamous cell carcinoma (HNSCC)

## Abstract

Growing evidence shows that hydroxamate-based compounds exhibit broad-spectrum pharmacological properties including anti-tumor activity. However, the precise mechanisms underlying hydroxamate derivative-induced cancer cell death remain incomplete understood. In this study, we explored the anti-tumor mechanisms of a novel aliphatic hydroxamate-based compound, WMJ-J-09, in FaDu head and neck squamous cell carcinoma (HNSCC) cells. WMJ-J-09 induced G2/M cell cycle arrest and apoptosis in FaDu cells. These actions were associated with liver kinase B1 (LKB1), AMP-activated protein kinase (AMPK) and p38 mitogen-activated protein kinase (p38MAPK) activation, transcription factor p63 phosphorylation, as well as modulation of p21 and survivin. LKB1-AMPK-p38MAPK signaling blockade reduced WMJ-J-09’s enhancing effects in p63 phosphorylation, p21 elevation and survivin reduction. Moreover, WMJ-J-09 caused an increase in α-tubulin acetylation and interfered with microtubule assembly. Furthermore, WMJ-J-09 suppressed the growth of subcutaneous FaDu xenografts *in vivo*. Taken together, WMJ-J-09-induced FaDu cell death may involve LKB1-AMPK-p38MAPK-p63-survivin signaling cascade. HDACs inhibition and disruption of microtubule assembly may also contribute to WMJ-J-09’s actions in FaDu cells. This study suggests that WMJ-J-09 may be a potential lead compound and warrant the clinical development in the treatment of HNSCC.

## Introduction

Head and neck squamous cell carcinomas (HNSCCs) represent the third and the fifth most common malignancy in Asian and Western countries, respectively (Ferlay et al., 2015). They account for approximately 550,000 new cases and 380,000 deaths annually (Global Burden of Disease Cancer Collaboration et al., 2017). Treatment of HNSCC is typically determined by staging information and histological subtype. Approximately two-thirds of patients diagnosed with HNSCC, however, present with advanced disease that carries poorer prognosis and lower over-all survival rate (Malumbres and Barbacid, 2009; Ran et al., 2015; Sacco and Cohen, 2015). As a result, there is an urgent need to develop novel agents or strategies to improve therapeutic outcome of HNSCC.

Survivin, the smallest member of the inhibitor of apoptosis protein (IAP) family, regulates cellular homeostasis with functions beyond inhibiting apoptosis (Srinivasula and Ashwell, 2008). It underlies various cellular events such as mitosis (Altieri, 2008), cell migration, angiogenesis, and metastasis (Ryan et al., 2009; Mehrotra et al., 2010; Altieri, 2013). Survivin is undetectable in most normal adult tissues with notable exceptions of hematopoietic or vascular endothelial cells (Gurbuxani et al., 2005), but it is highly expressed in most human cancers including HNSCC. Its expression is associated with tumor progression and poor clinical outcome (Ambrosini et al., 1997; Altieri, 2003a,b, 2008; Hunter et al., 2007; Rodriguez-Berriguete et al., 2015). Survivin thus represents a promising therapeutic target for cancer treatment (Altieri, 2003b, 2008; Shirai et al., 2009). Survivin gene expression is primarily regulated at the transcriptional level. Transcription factors including signal transducer and activator of transcription 3 (STAT3) (Chuang et al., 2017), hypoxia-inducible factor-1α (HIF-1α) and specificity protein 1 (Sp1) (Guha and Altieri, 2009) upregulate survivin gene expression. In contrast, tumor suppressor p53 and its related protein p63 may suppress survivin expression (Huang et al., 2015; Yen et al., 2016). While the abrogation of p53 signaling appears to be the main molecular abnormality in HNSCC, p63 is rarely deleted or mutated in most cancers including HNSCC (Soussi and Beroud, 2001). Therefore, pharmacological targeting of the p63-survivin cascade is a potential therapeutic strategy in HNSCC treatment.

Recent development in drug discovery has highlighted the key pharmacophore, hydroxamate, which exhibits diverse biological and pharmacological activities (Bertrand et al., 2013). Although the exact mechanisms remain unclear, there is growing evidence of aliphatic hydroxamate-based compounds’ potential as anti-inflammatory (Chen et al., 2015), anti-angiogenic (Chang et al., 2015) and anti-tumor agents (Huang et al., 2015; Chuang et al., 2017).

It is believed that abnormal HDACs expressions and alterations in cellular acetylation levels are mechanistically correlated with cancer pathogenesis (Bolden et al., 2006). For instance, HDACs inhibition is capable of inducing cell death in most human cancer cell lines, as well as suppressing tumor progression in preclinical models (Bolden et al., 2006; Falkenberg and Johnstone, 2014). Most HDAC inhibitors identified to date are hydroxamate derivatives, including the three novel HDAC inhibitors, vorinostat (suberoylanilide hydroxamate, SAHA) (Grant et al., 2007), belinostat (PXD101) (Poole, 2014) and panobinostat (LBH589) (Cheng et al., 2015) which have been approved by the U.S. Food and Drug Administration (FDA) for the treatment of various hematologic malignancies. Abexinostat, another hydroxamate-based compound, also exhibits anti-tumor properties and is currently in clinical trials (Aggarwal et al., 2017). These findings suggest that additional hydroxamate-based compounds may exhibit anti-tumor activities capable of therapeutic applications, and are worth further development. We synthesized a series of aliphatic hydroxamate-based compounds, the WMJ-J compounds, and investigated their anti-HNSCC properties in an effort to develop novel anti-tumor hydroxamate-based compounds. Among these compounds, WMJ-J-09 displayed enhancing effects in inducing HNSCC cell death. This study aims to explore the underlying mechanisms of WMJ-J-09 in inducing cell death in FaDu human HNSCC-derived cancer cells.

## Materials and Methods

### Reagents

Transfection reagent, TurboFect^TM^, MEM and DMEM/F12 medium, FBS, TrypLE^TM^ and all cell culture reagents were purchased from Invitrogen (Carlsbad, CA, United States). MTT was from Sigma–Aldrich (St. Louis, MO, United States). SB203580, colchicine, paclitaxel, and mithramycin A were purchased from Calbiochem (San Diego, CA, United States). Antibodies against normal IgG, Sp1 and p21 were purchased from Santa Cruz Biotechnology (Santa Cruz, CA, United States). Antibodies against p63, p63 phosphorylated at Ser160/162, LKB1, LKB1 phosphorylated at Ser428, p38MAPK, p38MAPK phosphorylated at Thr180/Tyr182, AMPKα, AMPKα phosphorylated at Thr172, STAT3, STAT3 phosphorylated at Tyr705, survivin, caspase 3 active form and PARP were from Cell Signaling Technology (Danvers, MA, United States). Anti-mouse and anti-rabbit IgG conjugated HRP antibodies, as well as antibodies against cyclin D1, α-tubulin, GAPDH, Myc tag and DDDDK (Flag) were obtained from GeneTex, Inc. (Irvine, CA, United States). The enhanced chemiluminescence detection kit was from EMD Millipore (Billerica, MA, United States). All materials for immunoblotting were purchased from Bio-Rad (Hercules, CA, United States). HDAC8-Flag (Addgene plasmid # 13825) (Waltregny et al., 2005) construct was kindly provided by Dr. Eric Verdin (Department of Medicine, University of California, San Francisco, United States). The construct pcDNA-HDAC6-FLAG (Kawaguchi et al., 2003) was a gift from Tso-Pang Yao (Addgene plasmid # 30482). Dr. Morris Birnbaum (HHMI, Philadelphia, PA, United States) kindly provided the construct AMPK dominant negative mutant (AMPK-DN). Survivin promoter luciferase construct (survivin-luc) was purchased from Health Research, Inc. (Roswell Park Cancer Institute Division, Buffalo, NY, United States). Renilla-luc and the Dual-Glo luciferase assay system were purchased from Promega (Madison, WI, United States). All other chemicals were obtained from Sigma–Aldrich (St. Louis, MO, United States).

### Synthesis of WMJ-J Compounds

WMJ-J compounds were synthesized as described in the Supplementary Information.

### Cell Culture

Two human HNSCC cell lines, FaDu hypopharyngeal carcinoma cell line and SCC25 tongue squamous cell carcinoma cell line, were obtained from the Bioresource Collection and Research Center (BBRC, Hsinchu, Taiwan). Another human HNSCC cell line, SCC9 tongue squamous cell carcinoma cell line, was obtained from the American Type Culture Collection (Livingston, MT, United States). FaDu cells were maintained in MEM medium containing 10% FBS, 1 mM sodium pyruvate, 100 μg/ml streptomycin and 100 U/ml penicillin G in a humidified 37°C incubator. SCC25 and SCC9 cells were maintained in DMEM/F12 medium containing 10% FBS, 100 μg/ml streptomycin, 100 U/ml penicillin G, and 400 ng/ml hydrocortisone in a humidified 37°C incubator.

### Cell Viability (MTT) Assay

Cell viability was determined by the colorimetric 3-(4,5-dimethylthiazol-2-yl)-2,5-diphenyl tetrazolium bromide (MTT) assay as described previously (Chen et al., 2015).

### Flow Cytometry

FaDu cells were treated with WMJ-J-09 at indicated concentrations for 24 or 48 h. Cells were washed twice with PBS and fixed in 70% ethanol at 0°C for another 24 h. After washed with phosphate-citric acid buffer, cells were stained by staining buffer (20 μg/ml PI, 100 μg/ml RNase A and 0.1% Triton X-100) in the dark for 30 min. Flow cytometry was performed using the FACScan and CellQuest program (BD Biosciences, San Jose, CA, United States). The percentage of PI-stained cells in the subG1 (Apoptosis, Apo), G0/G1, S or G2/M region was analyzed using the ModFit (BD Biosciences, San Jose, CA, United States) or FCS Express (De Novo Software, Glendale, CA, United States) program.

### Immunoblotting

Cells were harvested in lysis buffer [140 mM NaCl, 0.5% NP-40, 10 mM Tris (pH 7.0), 0.2 mM leupeptin, 0.05 mM pepstatin A and 2 mM PMSF]. Equal amounts of protein samples were subjected to SDS–PAGE and transferred onto a NC membrane (Pall Corporation, Port Washington, NY, United States). After blocking in a 5% non-fat milk-containing blocking buffer for 1 h, proteins were recognized using specific primary antibodies followed by HRP-conjugated secondary antibodies. To detect immunoreactivity, enhanced chemiluminescence was used according to manufacturer’s instructions. A computing densitometer with a scientific imaging system (BioSpectrum AC System, UVP) was employed to obtain the quantitative data.

### Transfection in FaDu Cells

FaDu cells (7 × 10^4^ cells per well) were transfected with survivin promoter-luc (survivin-luc) or Sp1-luc plus renilla-luc for reporter assay or transfected with pcDNA, AMPK-DN, HDAC3-Flag, HDAC4-Flag, HDAC6-Flag or HDAC8-Flag for immunoblotting using TurboFect Transfection Reagent (Invitrogen, Carlsbad, CA, United States) according to manufacturer’s instructions.

### Dual-Glo Luciferase Assay (Reporter Assay)

After transfection with reporter constructs plus renilla-luc, FaDu cells with or without treatments were harvested. The luciferase reporter activity was determined using a Dual-Glo luciferase assay system kit (Promega, Madison, WI, United States) according to manufacturer’s instructions, and was normalized based on renilla luciferase activity.

### Suppression of p63 and LKB1 Expression

Target gene suppression was performed as previously described (Chuang et al., 2017). For *p63* or *lkb1* suppression, pre-designed siRNAs targeting the human *p63* or *lkb1* and negative control siRNA were purchased from Sigma–Aldrich (St. Louis, MO, United States). The siRNA oligonucleotides were as follows: negative control siRNA, 5′-gaucauacgugcgaucaga-3′; *p63* siRNA, 5′-ggaugaaccgccguccaau-3′ and *lkb1* siRNA, 5′-aaucagcugacagaaguac-3′.

### Immunofluorescence Microscopy

To determine tubulin distribution, FaDu cells were seeded on glass cover slips for 24 h. Cells were treated with WMJ-J-09, paclitaxel or colchicine for another 24 h. Cells were then washed twice with PBS and fixed in 4% paraformaldehyde in PBS for 15 min at room temperature. After permeabilization for 30 min in 0.1% Triton X-100 in PBS, FaDu cells were washed twice and incubated with 1% BSA in PBS for another 1 h. To observe tubulin distribution, cells were reacted with rabbit anti-β-tubulin antibody (Cell Signaling Technology, Danvers, MA, United States) (1:100 dilution in PBS) for 16 h at 4°C. Slides were washed twice and incubated with FITC-conjugated goat anti-rabbit IgG for another 1 h. Slides were mounted with DAPI containing mounting solution (SlowFad Gold, Thermo Fisher Scientific, Waltham, MA, United States) and then observed under a confocal microscope (Zeiss, LSM 410). Green fluorescence indicated β-tubulin, and blue fluorescence (derived from DAPI) represented nuclei.

### Reverse-Transcription-Quantitative Real-Time Polymerase Chain Reaction (RT-qPCR)

FaDu cells with or without treatments were harvested and total RNA was isolated for complementary DNA (cDNA) synthesis as described previously (Chuang et al., 2017). Real time PCR was performed with the GoTaq qPCR Master Mix (Promega, Madison, WI, United States) using StepOne Real-Time PCR systems (Applied Biosystems, Grand Island, NY, United States). The cycling conditions were: hot-start activation at 95°C for 2 min, followed by 40 cycles of denaturation at 95°C for 15 s, annealing/extension at 60°C for 60 s, respectively. Primer pairs for the transcripts of survivin and GAPDH are: survivin sense, 5′-gcc ttt cct taa agg cca tc-3′; survivin anti-sense, 5′-aac cct tcc cag act cca ct-3′; GAPDH sense, 5′-gtc agt ggt gg acct gac ct-3′; GAPDH anti-sense, 5′-agg ggt cta cat ggc aac tg-3′.

### Ethics Statement

This study was carried out in strict accordance with the recommendations in the Guide for the Care and Use of Laboratory Animals of the National Institutes of Health (NIH publication no. 85-23, revised 1996). The protocols described below were also approved by the Taipei Medical University Laboratory Animal Care and Use Committee (Permit Number: LAC-2015-0215).

### Mouse Xenograft Model

Animal studies are reported in accordance with the ARRIVE guidelines (Kilkenny et al., 2010; McGrath and Lilley, 2015). The xenograft model with nude_nu/nu_ mice as described previously (Yen et al., 2016) was employed to determine WMJ-J-09’s *in vivo* anti-tumor effects. Four-week old male nude_nu/nu_ mice with body weight about 25 g were obtained from BioLasco (Taipei, Taiwan) and used for the experiment presented in **Figure 8**. All the mice were housed (three mice per cage) in clean specific pathogen free (SPF) rooms (standard 12-h light/12-h dark cycle at 22°C) in Laboratory Animal Center of Taipei Medical University, and maintained on standard chow and autoclaved water. All mice were randomly allocated to individually ventilated cage (IVC) by vivarium staff, upon transfer from BioLASCO into the animal housing room. All mice purchased from BioLASCO were acclimatized in the animal housing room for 7 days prior to starting experiments. FaDu cells were harvested and resuspended in PBS, and 5 × 10^6^ cells in a volume of 250 μl were injected subcutaneously into the flank of each mouse. Once the tumor reached approximately 150 mm^3^, animals were randomized into the control group (six mice) and the treatment group (six mice), which received WMJ-J-09 20 mg/kg/day. The treatment was administered intraperitoneally once daily for 23 days. Tumors were measured every day by a digital caliper. Tumor volume was calculated using the formula V $({\text{mm}}^{3})=[{\mathrm{ab}}^{2}]\times 0.52$, where *a* is the length and *b* is the width of the tumor (Chang et al., 2015). The body weights of the nude mice were examined daily within 23 days treatment of vehicle or WMJ-8-B. At the end of treatment, animals were sacrificed by carbon dioxide euthanasia and tumors were removed and weighed. The study conforms to the Guide for the Care and Use of Laboratory Animals (NIH publication No. 85-23, revised 1996) and was approved by the Taipei Medical University Animal Care and Use Committee.

### Randomization and Blinding

The same cell (FaDu cell) was used to evaluate the effects of WMJ-J-09 versus the related control in every single experiment. Therefore, formal randomization was not employed. Mice used in xenograft model were randomly allocated to cages by vivarium staff and randomized into vehicle- or WMJ-J-09-treated group before the treatment. In addition, we have different people conducting experiments (operator) and analyzing data (analyst) for blinding.

### Data and Statistical Analysis

In the present study, the data and statistical analysis comply with the recommendations on experimental design and analysis in pharmacology (Curtis et al., 2015). Results are expressed as mean ± standard error of mean (SEM) (*n* ≥ 5), where ‘*n’* refers to independent values, and not replicates. Normalization was performed to compare the differences after the treatment to control for unwanted sources of variation and to reveal relevant trends. Statistical analysis was performed using SigmaPlot 10 (Build 10.0.0.54; Systat Software, San Jose, CA, United States). Statistical comparisons between two groups were evaluated by the unpaired Student’s *t*-test for parametric analysis or Mann–Whitney test for non-parametric analysis. Statistical comparisons among more than two groups were evaluated by one-way analysis of variance (ANOVA) with Tukey’s *post hoc* test for parametric analysis or Kruskal–Wallis test followed by Dunn’s multiple comparisons for non-parametric analysis. *Post hoc* tests were run only if F achieved *P* < 0.05 and there was no significant inhomogeneity. A *P*-value smaller than 0.05 was defined as statistically significant.

## Results

### WMJ-J-09 Caused Cell Cycle Arrest and Apoptosis in FaDu Cells

**FIGURE 1:**
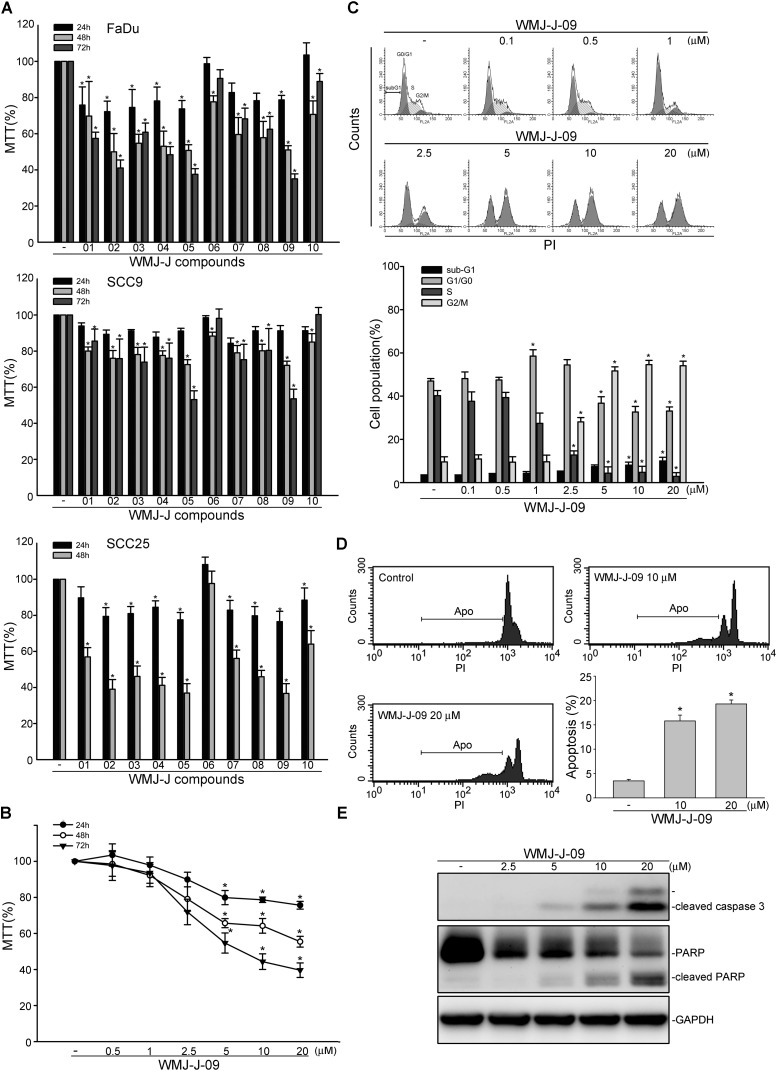
WMJ-J-09 caused G2/M cell cycle arrest and apoptosis in FaDu cells. **(A)** FaDu, SCC9, and SCC25 cells were treated with vehicle or WMJ-J-01∼10 at 10 μM for 24, 48, or 72 h. Cell viability was determined by an MTT assay. Each column represents the mean ± SEM of five independent experiments performed in duplicate (Statistically significant differences were determined using the Kruskal–Wallis test. ^∗^*p* < 0.05, compared with the control group). Technical replicates were used to ensure the reliability of singe values for each experiment. **(B)** FaDu cells were treated with vehicle or WMJ-J-09 at indicated concentrations for 24, 48, or 72 h. Cell viability was determined by an MTT assay. Each column represents the mean ± SEM of six independent experiments performed in duplicate (Statistically significant differences were determined using the Kruskal–Wallis test. ^∗^*p* < 0.05, compared with the control group). **(C)** Cells were treated with vehicle or WMJ-J-09 at indicated concentrations for 24 h. The percentage of propidium iodide-stained cells in sub-G1, G0/G1, S, and G2/M phases was analyzed by flow cytometric analysis as described in the Section “Materials and Methods.” Each column represents the mean ± SEM of five independent experiments (Statistically significant differences were determined using one-way ANOVA, with Tukey’s *post hoc* test. ^∗^*p* < 0.05, compared with the control group). **(D)** Cells were treated with vehicle or WMJ-J-09 at indicated concentrations for 48 h. The percentage of PI-stained cells in apoptosis (Apo, subG1) region was analyzed by flow cytometric analysis as described in the Section “Materials and Methods.” Each column represents the mean ± SEM of six independent experiments (Statistically significant differences were determined using one-way ANOVA, with Tukey’s *post hoc* test. ^∗^*p* < 0.05, compared with the control group). **(E)** Cells were treated with WMJ-J-09 at indicated concentrations for 48 h. The extent of cleaved caspase 3 and PARP were determined by immunoblotting. Typical traces shown are representative of four independent experiments.

3-[4, 5-dimethylthiazol-2-yl]-2, 5-diphenyltetrazolium bromide assay was employed to assess the effects of WMJ-J compounds (WMJ-J-01∼10) (Supplementary Figure S1) on cell viability in FaDu, SCC9, and SCC25 human HNSCC cell lines. As shown in **Figure 1A**, these WMJ-J compounds at 10 μM are all effective in decreasing cell viability in FaDu, SCC9, and SCC25 cells after 24, 48, or 72 h exposure. Among these compounds, WMJ-J-09 exhibits the most potent cytotoxic effects in these three HNSCC cell lines (**Figure 1A**). Therefore, we sought to explore the underlying mechanisms by which WMJ-J-09 induces FaDu cell death in the following experiments. Results from MTT assay further showed that WMJ-J-09 time- and concentration-dependently decreased cell viability in FaDu cells (**Figure 1B**). To determine whether WMJ-J-09 affects cell cycle progression or induces apoptosis, flow cytometry with PI labeling was employed. As shown in **Figure 1C**, treatment of cells with WMJ-J-09 for 24 h increased the percentage of PI-stained cells in the G2/M region. This effect was accompanied by a concomitant decrease in the percentage of PI-stained cells in the S region (**Figure 1C**). Longer exposure to WMJ-J-09 (48 h) further increased the percentage of PI-stained cells in the apoptosis (Apo, sub-G1) region (**Figure 1D**). Results from immunoblotting analysis also showed that WMJ-J-09 exposure led to an increase in the cleaved (active) forms of caspase 3 and the selective caspase 3 substrate, PARP (**Figure 1E**). Together these findings indicate that WMJ-J-09 induces G2/M cell cycle arrest and apoptosis in FaDu HNSCC cells.

### WMJ-J-09 Induced p21^cip/Waf1^ Induction and Survivin Reduction in FaDu Cells

**FIGURE 2:**
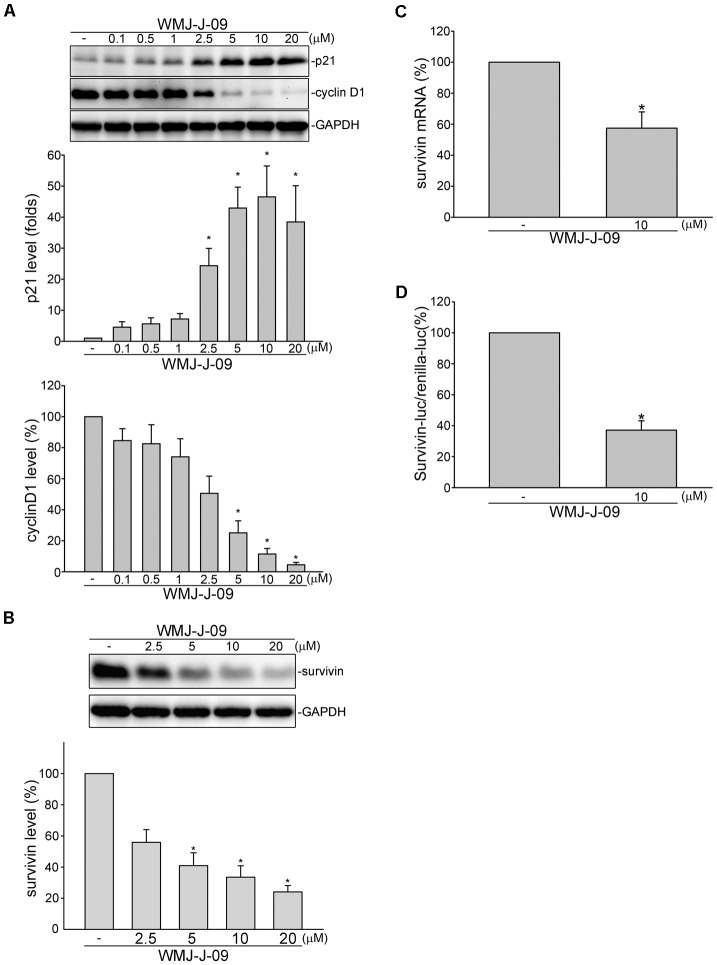
WMJ-J-09 reduced survivin expression in FaDu cells. **(A)** FaDu cells were treated with vehicle or WMJ-J-09 at indicated concentrations for 24 h. Protein levels of p21^cip/Waf^ and cyclin D1 were determined by immunoblotting. Each column represents the mean ± SEM of eight independent experiments (Statistically significant differences were determined using the Kruskal–Wallis test. ^∗^*p* < 0.05, compared with the control group). **(B)** Cells were treated with vehicle or WMJ-J-09 at indicated concentrations for 24 h. Protein level of survivin was determined by immunoblotting. Each column represents the mean ± SEM of six independent experiments (Statistically significant differences were determined using the Kruskal–Wallis test. ^∗^*p* < 0.05, compared with the control group). **(C)** Cells were treated with vehicle or WMJ-J-09 at 10 μM for 6 h. The survivin mRNA level was determined by an RT-qPCR as described in the Section “Materials and Methods.” Each column represents the mean ± SEM of five independent experiments (Statistically significant differences were determined using the Mann–Whitney test. ^∗^*p* < 0.05, compared with the control group). **(D)** Cells were transiently transfected with survivin promoter reporter construct (–3569/+1, survivin-luc) and renilla-luc for 24 h followed by the treatment with WMJ-J-09 at 10 μM for another 24 h. Reporter assay was performed as described in the Section “Materials and Methods.” Each column represents the mean ± SEM of five independent experiments performed in duplicate (Statistically significant differences were determined using the Mann–Whitney test. ^∗^*p* < 0.05, compared with the control group).

Cyclin-dependent kinase (CDK) inhibitor p21^cip/Waf1^, cyclin D1 and survivin play essential roles in regulating cell cycle progression. We demonstrated recently that survivin reduction by s*urvivin* siRNA caused FaDu cell cycle arrest and death (Yen et al., 2016). We thus examined whether WMJ-J-09 had any effects on these proteins in FaDu cells. As shown in **Figure 2A**, treatment of cells with WMJ-J-09 significantly elevated p21^cip/Waf1^ protein levels, accompanied by decreased cyclin D1 levels. Exposure to WMJ-J-09 also led to significant decreases in survivin protein (**Figure 2B**) and mRNA (**Figure 2C**) levels. Similar effects on p21^cip/Waf^ and survivin were also observed in another HNSCC cell line, SCC25, after WMJ-J-09 exposure (Supplementary Figure S2). Moreover, WMJ-J-09 reduced survivin-promoter luciferase activity as determined by reporter assay with human survivin-promoter reporter construct (-3569/+1, survivin-luc) (**Figure 2D**). It appears that WMJ-J-09 might negatively regulate survivin expression at the transcriptional level. These results suggest that WMJ-J-09-induced FaDu cell death might involve p21^cip/Waf1^ elevation and survivin reduction.

### p63 Contributes to WMJ-J-09’s Actions in FaDu Cells

**FIGURE 3:**
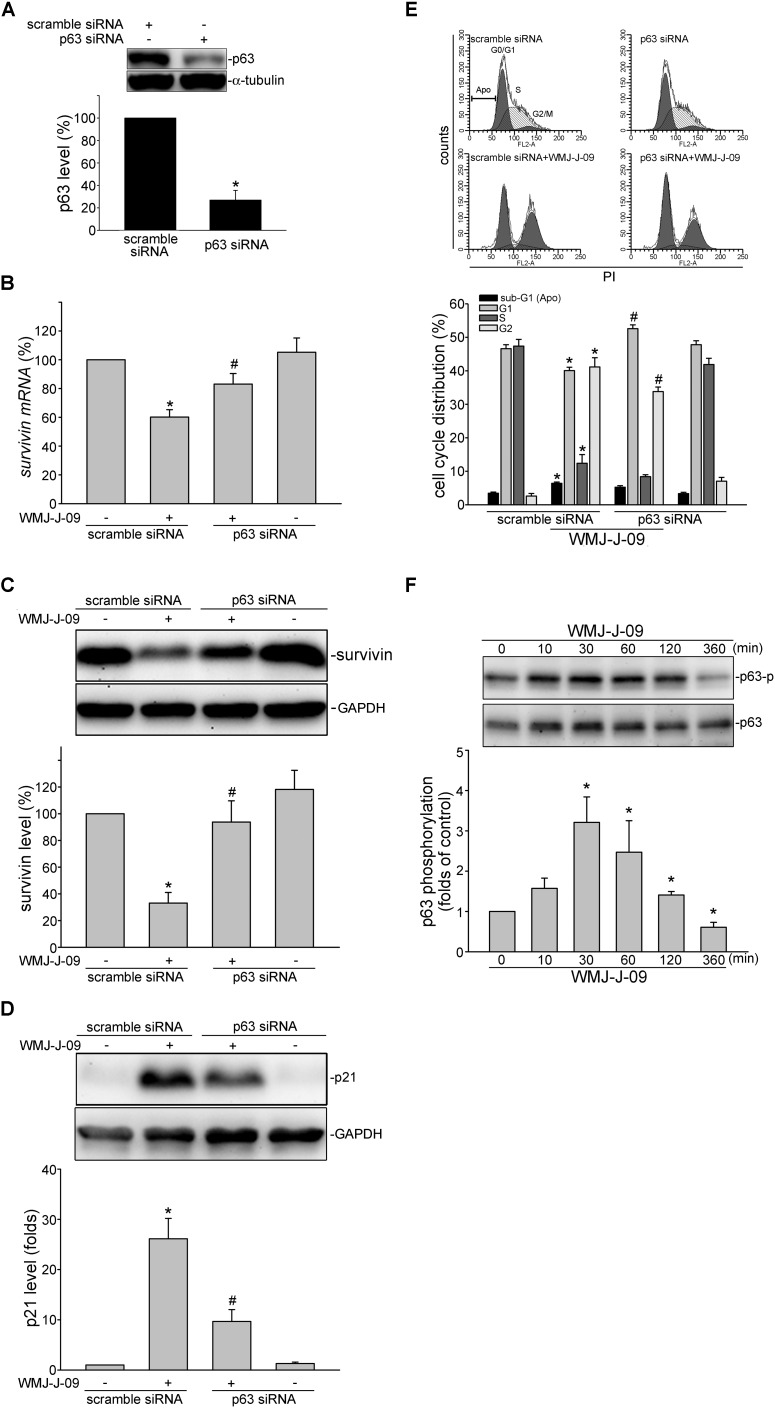
p63 contributes to WMJ-J-09-induced survivin reduction in FaDu cells. **(A)** FaDu cells were transfected with negative control siRNA or p63 siRNA for 48 h. p63 and α-tubulin levels were determined by immunoblotting. Each column represents the mean ± SEM of seven independent experiments (Statistically significant differences were determined using the Mann–Whitney test. ^∗^*p* < 0.05, compared with the vehicle-treated control group). **(B)** Cells were transfected as described in **A**. After transfection, cells were treated with vehicle or WMJ-J-09 at 10 μM for another 6 h. The survivin mRNA level was determined by an RT-qPCR as described in the Section “Materials and Methods.” Each column represents the mean ± SEM of six independent experiments (Statistically significant differences were determined using the Mann–Whitney test. ^∗^*p* < 0.05, compared with the vehicle-treated control group; ^#^*p* < 0.05, compared with the group treated with WMJ-J-09 alone) Cells were transfected as described in **A**. After transfection, cells were treated with vehicle or WMJ-J-09 at 10 μM for another 24 h. Protein levels of survivin **(C)** or p21^cip/Waf^ **(D)** were determined by immunoblotting. Each column represents the mean ± SEM of six independent experiments (Statistically significant differences were determined using the Mann–Whitney test. ^∗^*p* < 0.05, compared with the vehicle-treated control group; ^#^*p* < 0.05, compared with the group treated with WMJ-J-09 alone). **(E)** Cells were transfected as described in **A**. After transfection, cells were treated with vehicle or WMJ-J-09 at 10 μM for another 24 h. Flow-cytometric analysis was used to determine cell cycle distribution. Each column represents the mean ± SEM of five independent experiments (Statistically significant differences were determined using the Mann–Whitney test. ^∗^*p* < 0.05, compared with the vehicle-treated control group; ^#^*p* < 0.05, compared with the group treated with WMJ-J-09 alone). **(F)** Cells were treated with vehicle or WMJ-J-09 at 10 μM for indicated periods. The phosphorylation status of p63 was determined by immunoblotting. Each column represents the mean ± SEM of five independent experiments (Statistically significant differences were determined using the Kruskal–Wallis test. ^∗^*p* < 0.05, compared with the control group).

We next explored the underlying mechanisms by which WMJ-J-09 represses survivin expression. Transcription factor p63, a p53 family member, exhibits anti-proliferative and apoptotic properties via regulating p53-responsive target genes. p63 could activate p21^cip/Waf1^ promoter to up-regulate p21^cip/Waf1^ expression (Katoh et al., 2000). In contrast, p63 might counteract Sp1 binding to the promoter region and, thereby, suppress survivin expression (Hsu et al., 2012). FaDu cell is a p53-deficient HNSCC cell line (Kim et al., 1993) and p53-mediated apoptotic response has been reported defect in FaDu cells (Ndoye et al., 2004). Therefore, we explored whether p63 contributes to WMJ-J-09’s actions in FaDu cells. As shown in **Figure 3A**, transfection of cells with p63 siRNA markedly reduced the basal p63 level in FaDu cells. WMJ-J-09’s enhancing effects in reducing survivin mRNA (**Figure 3B**) and protein (**Figure 3C**) levels were significantly restored in cells transfected with p63 siRNA. p63 siRNA also inhibited WMJ-J-09-induced p21^cip/Waf1^ elevation (**Figure 3D**). Results from flow cytometric analysis further showed that WMJ-J-09-induced G2/M cell cycle arrest was suppressed in the presence of p63 siRNA (**Figure 3E**). Activation of p63, similar to p53, is regulated by its modifications such as phosphorylation (Finlan and Hupp, 2007). We used anti-phosphorylated p63 antibody (Cell Signaling Technology) to determine whether WMJ-J-09 induces p63 phosphorylation. As shown in **Figure 3F**, WMJ-J-09 significantly caused an increase in p63 phosphorylation in FaDu cells. These results indicate that WMJ-J-09’s impact on cell death involves, at least in part, p63-mediated survivin reduction.

### LKB1-AMPK-p38MAPK Signaling Mediated WMJ-J-09-Induced p63 Phosphorylation and Survivin Reduction in FaDu Cells

**FIGURE 4:**
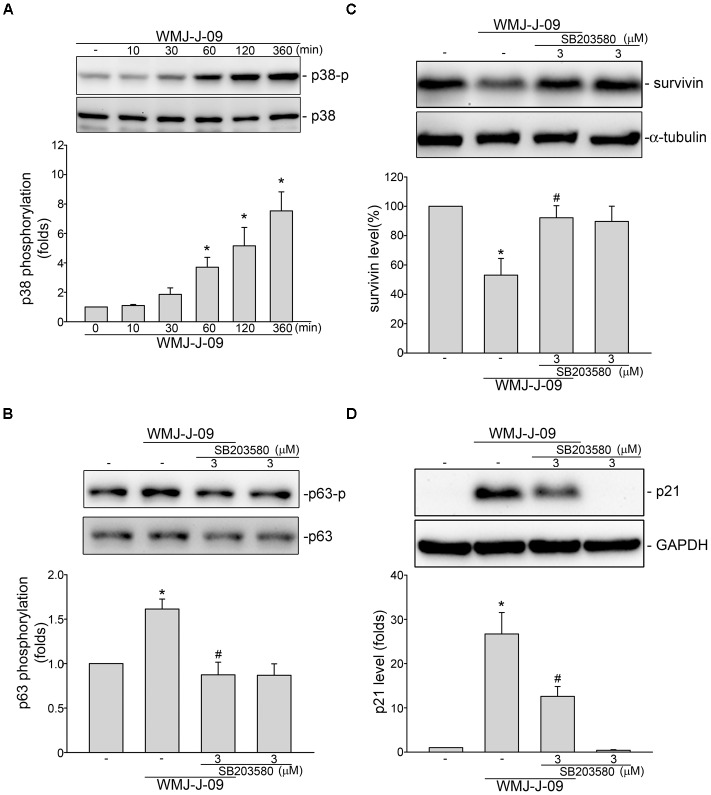
p38MAPK contributes to WMJ-J-09-induced p63 phosphorylation, survivin reduction and p21^cip/Waf^ elevation in FaDu cells. **(A)** Cells were treated with vehicle or WMJ-J-09 at 10 μM for indicated periods. The extent of p38MAPK phosphorylation was determined by immunoblotting. Each column represents the mean ± SEM of five independent experiments (Statistically significant differences were determined using the Kruskal–Wallis test. ^∗^*p* < 0.05, compared with the control group). **(B)** Cells were pretreated with p38MAPK inhibitor SB203580 at 3 μM for 30 min. After treatment, cells were stimulated with WMJ-J-09 at 10 μM for another 1 h. The extent of p63 phosphorylation was determined by immunoblotting. Each column represents the mean ± SEM of six independent experiments (Statistically significant differences were determined using the Mann–Whitney test. ^∗^*p* < 0.05, compared with the vehicle-treated control group; ^#^*p* < 0.05, compared with the group treated with WMJ-J-09 alone). Cells were pretreated with p38MAPK inhibitor SB203580 at 3 μM for 30 min. After treatment, cells were treated with WMJ-J-09 at 10 μM for another 24 h. Protein levels of survivin **(C)** or p21^cip/Waf^ **(D)** were determined by immunoblotting. Each column represents the mean ± SEM of five independent experiments (Statistically significant differences were determined using the Mann–Whitney test. ^∗^*p* < 0.05, compared with the vehicle-treated control group; ^#^*p* < 0.05, compared with the group treated with WMJ-J-09 alone).

**FIGURE 5:**
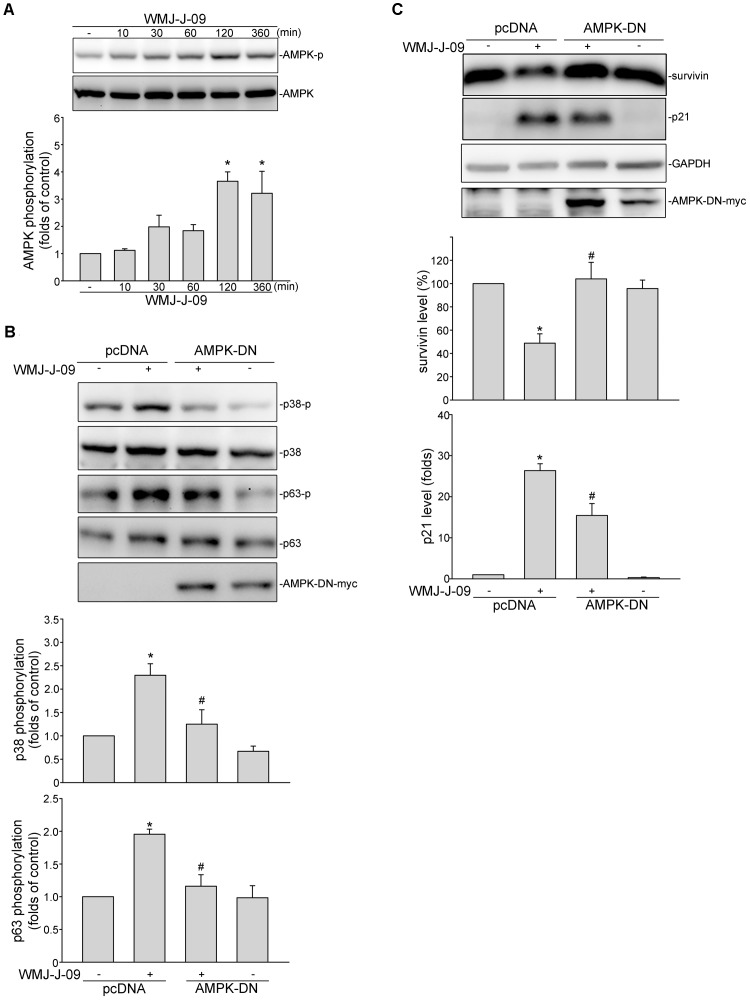
AMP-activated protein kinase (AMPK) mediates WMJ-J-09-induced p38MAPK and p63 phosphorylation, survivin reduction and p21^cip/Waf^ elevation in FaDu cells. **(A)** Cells were treated with vehicle or WMJ-J-09 at 10 μM for indicated periods. The extent of AMPK phosphorylation was determined by immunoblotting. Each column represents the mean ± SEM of five independent experiments (Statistically significant differences were determined using the Kruskal–Wallis test. ^∗^*p* < 0.05, compared with the control group). **(B)** Cells were transfected with pcDNA or AMPK-DN for 48 h. After transfection, cells were treated with vehicle or WMJ-J-09 at 10 μM for another 1 h. The extent of p38MAPK or p63 phosphorylation was determined by immunoblotting. Each column represents the mean ± SEM of five independent experiments (Statistically significant differences were determined using the Mann–Whitney test. ^∗^*p* < 0.05, compared with the vehicle-treated control group; ^#^*p* < 0.05, compared with the group treated with WMJ-J-09 alone). **(C)** Cells were transfected with pcDNA or AMPK-DN for 48 h. After transfection, cells were treated with vehicle or WMJ-J-09 at 10 μM for another 24 h. Protein levels of survivin or p21^cip/Waf^ were determined by immunoblotting. Each column represents the mean ± SEM of five independent experiments (Statistically significant differences were determined using the Mann–Whitney test. ^∗^*p* < 0.05, compared with the vehicle-treated control group; ^#^*p* < 0.05, compared with the group treated with WMJ-J-09 alone).

**FIGURE 6:**
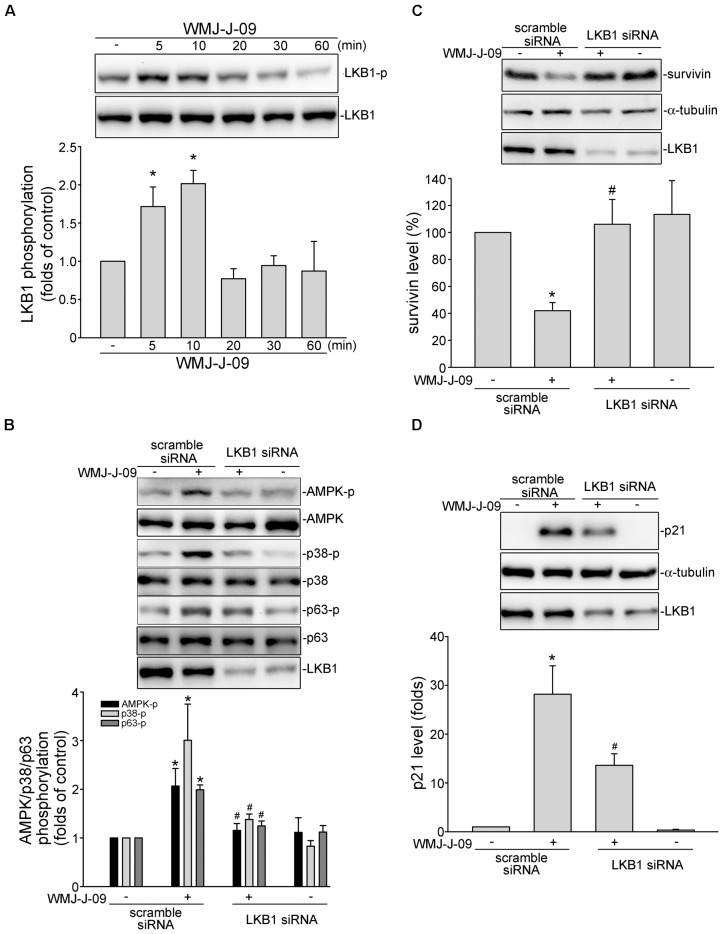
WMJ-J-09 activation of AMPK-p38MAPK-p63-survivin cascade involves LKB1 in FaDu cells. **(A)** Cells were treated with vehicle or WMJ-J-09 at 10 μM for indicated periods. The extent of LKB1 phosphorylation was determined by immunoblotting. Each column represents the mean ± SEM of six independent experiments (Statistically significant differences were determined using the Kruskal–Wallis test. ^∗^*p* < 0.05, compared with the control group). **(B)** Cells were transfected with negative control siRNA or LKB1 siRNA for 48 h. After transfection, cells were treated with vehicle or WMJ-J-09 at 10 μM for another 1 h. The phosphorylation status of AMPK, p38MAPK or p63 was determined by immunoblotting. Each column represents the mean ± SEM of six independent experiments (Statistically significant differences were determined using the Mann–Whitney test. ^∗^*p* < 0.05, compared with the vehicle-treated control group; ^#^*p* < 0.05, compared with the group treated with WMJ-J-09 alone). After transfection as described in **B**, cells were treated with vehicle or WMJ-J-09 at 10 μM for another 24 h. Protein levels of survivin **(C)** or p21^cip/Waf^ **(D)** were determined by immunoblotting. Each column represents the mean ± SEM of five independent experiments (Statistically significant differences were determined using the Mann–Whitney test. ^∗^*p* < 0.05, compared with the vehicle-treated control group; ^#^*p* < 0.05, compared with the group treated with WMJ-J-09 alone).

We next explored the signaling mechanisms underlying WMJ-J-09-induced p63 activation. Yen et al. (2016) has recently reported that p38MAPK activation leads to p63 phosphorylation and subsequent FaDu cell death. We thus examined whether p38MAPK contributes to WMJ-J-09’s actions in FaDu cells. As shown in **Figure 4A**, WMJ-J-09 caused an increase in p38MAPK phosphorylation in a time-dependent manner. SB203580, a pharmacological p38MAPK inhibitor, significantly inhibited WMJ-J-09-induced p63 phosphorylation (**Figure 4B**). SB203580 also reduced WMJ-J-09’s effects in inducing survivin reduction (**Figure 4C**) and p21^cip/Waf1^ elevation (**Figure 4D**) in FaDu cells. There is increasing evidence that serine/threonine kinase LKB1 regulates various cellular processes such as energy homeostasis, senescence, cell cycle arrest, and cell death (Tiainen et al., 2002; Lizcano et al., 2004; Vaahtomeri and Makela, 2011). A number of different mechanisms including AMPK activation (Luo et al., 2013) and p21^cip/Waf1^ induction (Tiainen et al., 2002) mediate these effects. In addition, AMPK-p38MAPK signaling cascade contributes to survivin reduction and subsequent cell death in p53-mutant HT29 colorectal cancer cells (Hsu et al., 2012). Therefore, we explored whether WMJ-J-09’s effects on FaDu cells involves LKB1 or AMPK signaling. As shown in **Figure 5A**, WMJ-J-09 time-dependently increased AMPK phosphorylation. Transfection of cells with AMPK dominant negative mutant (AMPK-DN) significantly inhibited WMJ-J-09-induced p38MAPK and p63 (**Figure 5B**) phosphorylation. AMPK-DN also suppressed the elevation of p21^cip/Waf1^ and restored the decrease of survivin in WMJ-J-09-stimulated cells (**Figure 5C**). Moreover, WMJ-J-09 caused an increase in LKB1 phosphorylation (**Figure 6A**). To establish the causal role of LKB1 in WMJ-J-09-induced activation of AMPK-p38MAPK-p63 cascade, LKB1 was silenced using siRNA strategy. As shown in **Figure 6B**, LKB1 knockdown by LKB1 siRNA significantly inhibited AMPK, p38MAPK, as well as p63 phosphorylation in FaDu cells after WMJ-J-09 exposure (**Figure 6B**). Furthermore, WMJ-J-09’s impacts on survivin (**Figure 6C**) and p21^cip/Waf1^ (**Figure 6D**) were reduced in the presence of LKB1 siRNA. Together these findings support the contention that WMJ-J-09 activates the LKB1-AMPK-p38MAPK-p63 signaling cascade to induce survivin reduction and p21^cip/Waf1^ elevation, resulting in FaDu cell death.

### WMJ-J-09 Induced α-Tubulin Acetylation and Disrupted Microtubule Assembly in FaDu Cells

**FIGURE 7:**
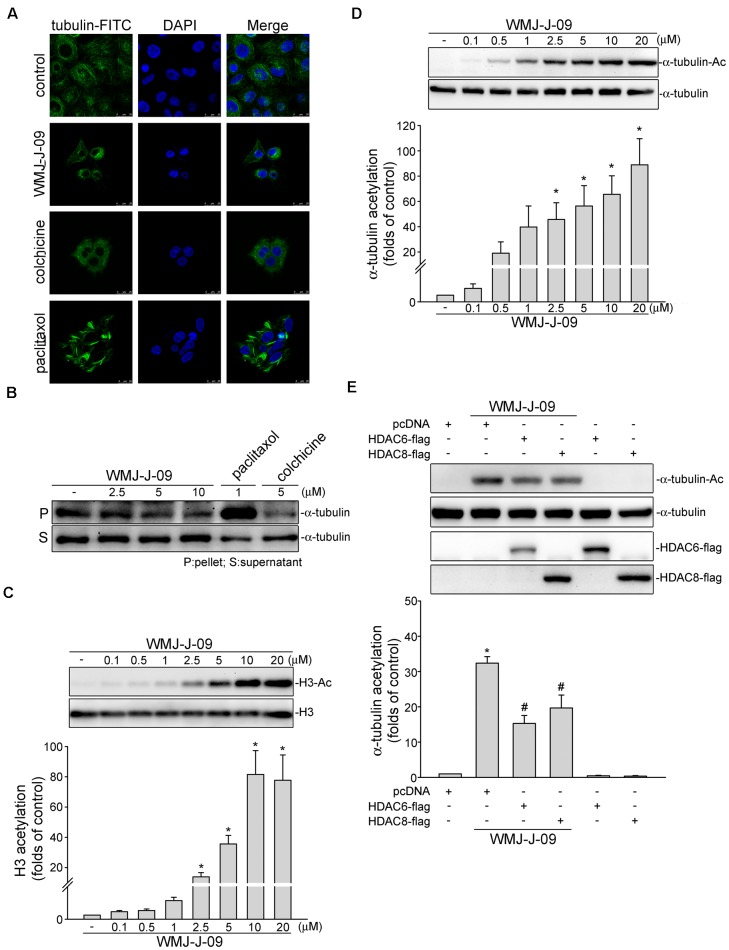
WMJ-J-09 induced α-tubulin acetylation and disrupted microtubule assembly in FaDu cells. **(A)** Cells were treated with the vehicle, WMJ-J-09 (10 μM), paclitaxel (1 μM), or colchicine (5 μM) for 24 h. Tubulin distribution was determined by immunofluorescence analysis as described in the Section “Materials and Methods.” Results shown are representative of five independent experiments. **(B)** Cells were treated with the vehicle, WMJ-J-09, paclitaxel, or colchicine at indicated concentrations for 24 h. After treatment, the fraction of polymerized tubulin was determined by immunoblotting. Typical traces shown are representative of five independent experiments. **(C)** Cells were treated with vehicle or WMJ-J-09 at indicated concentrations for 24 h. The acetylation status of histone 3 was determined by immunoblotting. Each column represents the mean ± SEM of seven independent experiments (Statistically significant differences were determined using the Kruskal–Wallis test. ^∗^*p* < 0.05, compared with the control group). **(D)** Cells were treated with WMJ-J-09 at indicated concentrations for 6 h. The acetylation status of α-tubulin was determined by immunoblotting. Each column represents the mean ± SEM of eight independent experiments (Statistically significant differences were determined using the Kruskal–Wallis test. ^∗^*p* < 0.05, compared with the control group). **(E)** Cells were transiently transfected for 48 h with pcDNA, HDAC6-flag, or HDAC8-flag. After transfection, cells were treated with WMJ-J-09 (5 μM) for another 6 h. The acetylation status of α-tubulin and flag-tagged HDAC6 and HDAC8 were determined by immunoblotting. Each column represents the mean ± SEM of six independent experiments [Statistically significant differences were determined using the Mann–Whitney test. ^∗^*p* < 0.05, compared with the pcDNA transfection (mock transfection) group treated with vehicle; ^#^*p* < 0.05, compared with the pcDNA transfection group treated with WMJ-J-09].

During mitosis, the integrity of cellular dynamic structures, microtubules, is essential for proper chromosome segregation. Interference with the assembly or disassembly of α- and β-tubulin into microtubules may cause G2/M cell cycle arrest and subsequent cell death (Jordan and Wilson, 2004). As described above, WMJ-J-09 caused G2/M cell cycle arrest in FaDu cells. We thus examined whether tubulin distribution is altered in WMJ-J-09-stimulated cells. The effects of two microtubule-targeting agents, colchicine and paclitaxel, on tubulin distribution in FaDu cells were also examined. As shown in **Figure 7A**, WMJ-J-09, similar to microtubule-disturbing agent colchicine, caused cellular microtubule depolymerization as determined by immunofluorescence microscopy analysis using specific antibodies against β-tubulin. In contrast, the microtubule-stabilizing agent paclitaxel induced microtubule polymerization in FaDu cells (**Figure 7A**). To further confirm that WMJ-J-09 is capable of disrupting microtubule assembly, cell extracts containing soluble (monomeric) or polymeric tubulin were prepared from FaDu cells after 24 h exposure to WMJ-J-09, paclitaxel or colchicine. As shown in **Figure 7B**, WMJ-J-09 or colchicine decreased the fraction of polymerized tubulin (in precipitate, pellets) as determined by immunoblotting. As expected, paclitaxel markedly increased the polymeric form of tubulin (**Figure 7B**). A number of studies have suggested that hydroxamate-based compounds might inhibit HDAC activity to cause cell death and suppress *in vivo* tumor growth (Rajak et al., 2014; West and Johnstone, 2014; Chuang et al., 2017). To assess whether WMJ-J-09 exhibits HDAC inhibitory properties, we examined the WMJ-J-09’s effects on histone 3 (H3) acetylation. As shown in **Figure 7C**, WMJ-J-09 significantly increased H3 acetylation in a concentration-dependent manner. It appears that WMJ-J-09 is capable of inhibiting HDACs in FaDu cells. Post-translational modifications of tubulin such as acetylation might interfere with microtubules assembly (Haggarty et al., 2003). We next explored whether WMJ-J-09 inhibition of HDACs results in α-tubulin acetylation. As shown in **Figure 7D**, WMJ-J-09 exposure for 6 h led to an increase in α-tubulin acetylation in a concentration-dependent manner. Furthermore, transfection of cells with HDAC6-Flag (a class II HDAC) or HDAC8-Flag (a class I HDAC) significantly inhibited WMJ-J-09-induced α-tubulin acetylation (**Figure 7E**). Together these results support a causal role of selective HDAC inhibition in WMJ-J-09-induced α-tubulin acetylation and subsequent disruption of microtubule assembly in FaDu cells.

### WMJ-J-09 Suppressed FaDu Tumor Xenograft Growth *in Vivo*

**FIGURE 8:**
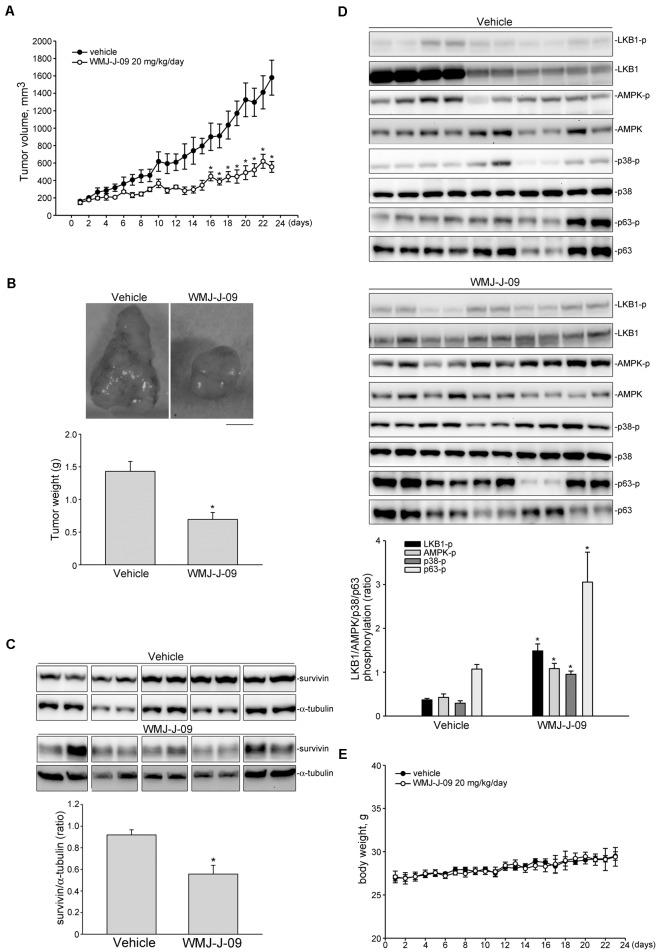
WMJ-J-09 suppressed FaDu tumor xenograft growth *in vivo*. **(A)** Nude mice bearing xenografts of FaDu cells were treated intraperitoneally with WMJ-J-09 20 mg/kg/day for 23 days. The control group received vehicle only. Tumor volumes were calculated as described in the Section “Materials and Methods.” Values represent the mean ± SEM, *n* = 6. **(B)** After 23 days of treatment, mice were sacrificed and tumors were dissected and weighted. Scale bar = 0.5 cm; Each column represents the mean ± SEM (Statistically significant differences were determined using the Student’s *t*-test. ^∗^*p* < 0.05 as compared with the vehicle-treated control group, *n* = 6). Protein lysates obtained from five randomly selected xenograft tumors were subjected to immunoblotting for assessing survivin **(C)** or phosphorylated LKB1, AMPK, p63 or STAT3 **(D)** levels. Values represents the mean ± SEM of five tumors from each group performed in duplicate (Statistically significant differences were determined using the Student’s *t*-test. ^∗^*p* < 0.05 as compared with the vehicle-treated control group). Technical replicates were used to ensure the reliability of single values for each experiment. **(E)** The body weights of the nude mice were examined daily within 23 days treatment of vehicle or WMJ-J-09. Values represent the mean ± SEM *n* = 6 for vehicle-treated control group and *n* = 6 for WMJ-J-09-treated group.

We next used a mouse xenograft model to determine the *in vivo* effects of WMJ-J-09. FaDu cells were subcutaneously injected into the right flank of nude mouse. After allowing the tumors to grow to an average size of approximately 150 mm^3^, either vehicle or WMJ-J-09 (20 mg/kg/day) was intraperitoneally administrated for 23 days. At the end of the 23-day treatment, mice were sacrificed and xenografts were collected. Comparing to the vehicle-treated control group, WMJ-J-09 suppressed FaDu tumor xenograft growth (**Figure 8A**) and reduced tumor weight (**Figure 8B**). We also determined the protein levels of survivin in the excised tumors. As shown in **Figure 8C**, survivin level was decreased in FaDu xenografts from WMJ-J-09-treated mice. We further examined the phosphorylation status of LKB1, AMPK, p38MAPK and p63 in the excised FaDu xenografts. As shown in **Figure 8D**, WMJ-J-09 significantly increased LKB1, AMPK, p38MAPK and p63 phosphorylation in FaDu xenografts. Injection of WMJ-J-09 at 20 mg/kg/day had no significant effects on mouse body weight as compared to the vehicle-treated control group within 23 days (**Figure 8E**). These results suggest that WMJ-J-09 treatment is capable of suppressing tumor growth *in vivo* through, at least in part, activation of LKB1-AMPK-p38MAPK-p63 cascade and survivin reduction.

## Discussion

Despite advances in knowledge of molecular alterations in HNSCC and advances in therapy, the 5-year survival rate of patients with HNSCC has not significantly improved over the past few decades. HNSCC remains one of the leading causes of cancer-related death, highlighting the need for novel therapeutic agents or strategies. Aliphatic hydroxamate-based compounds have the potential of contributing to the development of novel anti-cancer drugs, but the underlying mechanisms remain incompletely understood (Chang et al., 2015; Huang et al., 2015; Chuang et al., 2017).

In this study, we demonstrated that WMJ-J-09, a novel aliphatic hydroxamate-base compound, activates LKB1-AMPK-p38MAPK-p63-survivin and/or p21^cip/Waf^ cascade to cause G2/M cell cycle arrest and apoptosis in FaDu HNSCC cells. We also noted that WMJ-J-09 induces α-tubulin acetylation and disrupts microtubule assembly through HDACs inhibition. WMJ-J-09 also suppressed the growth of FaDu xenografts in *in vivo* models.

In keeping with previous reports that aliphatic hydroxamate derivatives elevate p21^cip/Waf^ level and inhibit cancer cell proliferation (Huang et al., 2015; Chuang et al., 2017), we showed that WMJ-J-09 elevates p21^cip/Waf^ levels, which was accompanied by decreased survivin levels, in FaDu and SCC25 cells. Survivin reduction leads to cell cycle arrest and apoptosis in FaDu cells (Yen et al., 2016). We noted that WMJ-J-09’s effect on survivin level was reduced in cells transfected with p63 siRNA. p63 siRNA also suppressed WMJ-J-09-induced G2/M cell cycle arrest. These observations indicate that p63’s down-regulation of survivin may account for WMJ-J-09’s anti-proliferative and apoptotic effects in FaDu cells.

Post-translational modifications, including phosphorylation, sumoylation and acetylation, regulate p63 stability and transcriptional activity (Finlan and Hupp, 2007). We demonstrated that WMJ-J-09 activates LKB1-AMPK-p38MAPK cascade resulting in p63 phosphorylation. WMJ-J-09 also exhibited inhibitory effect on HDACs, which raises the possibility that HDAC inhibition is an intermediate step by which WMJ-J-09 acetylates and activates p63 in FaDu cells. Trichostatin A, a hydroxamate-based HDAC inhibitor, has been shown to cause IκB kinase-β (IKKβ) dephosphorylation and subsequent C6 glioma cell death (Hsu et al., 2011). Moreover, Liao et al. (2013) recently reported that IKKβ augments p63 transcriptional activity through interference of the interaction between p63 and p300, a HAT. Whether WMJ-J-09 induces p63 acetylation and the underlying mechanisms need further investigations. The possibility of IKKβ contributing to WMJ-J-09-induced p63 activation in FaDu cells should also be explored.

In contrast to the negative regulatory role of p63 on survivin expression, Sp1 (Mityaev et al., 2008) and STAT3 (Chuang et al., 2017) activate survivin promoter to up-regulates its expression. Constitutive STAT3 Tyr705 phosphorylation and activation is found in most cancers such as breast cancer (Diaz et al., 2006; Gritsko et al., 2006; Leslie et al., 2006) and HNSCC (Geiger et al., 2016). In addition, aberrant STAT3 activation in HNSCC contributes to tumor growth and resistance to standard therapies (Masuda et al., 2002; Sen et al., 2012). We recently demonstrated that STAT3 knockdown by STAT3 siRNA causes survivin reduction and subsequent cell death in triple negative breast cancer (Chuang et al., 2017) and HNSCC (Yen et al., 2016) cells. We noted in this study that WMJ-J-09 induces STAT3 Tyr705 dephosphorylation and reduces its transcriptional activity as determined by reported assay in FaDu cells. WMJ-J-09 also significantly decreased STAT3 Tyr705 phosphorylation in the excised FaDu xenografts (Supplementary Figure S3). It appears that WMJ-J-09’s reduction of survivin also contributes to STAT3 inactivation in FaDu cells. The precise mechanisms by which WMJ-J-09 dephosphorylates STAT3 remain unresolved. We recently reported that HDACs inhibition leads to STAT3 dephosphorylation via protein tyrosine phosphatase SHP-1 activation in breast cancer cells (Chuang et al., 2017). It is likely that WMJ-J-09 activates a protein tyrosine phosphatase such as SHP-1 that dephosphorylates STAT3 and thereby downregulates survivin expression in FaDu cells. Whether WMJ-J-09-induced STAT3 inactivation involves HDACs inhibition and SHP-1 or other phosphatases needs to be further investigated. On the other hand, STAT3 inactivation may also occur via AMPK activation (Vasamsetti et al., 2015). Suppression of LKB1 and AMPK activity is essential for STAT3 activation in smooth muscle cells after arterial injury (Yu et al., 2012). It raises the possibility that WMJ-J-09 activation of LKB1-AMPK signaling cascade not only activates p63, but also inactivates STAT3 in FaDu cells. The link between AMPK-STAT3 and LKB1-AMPK-p38MAPK-p63 cascades and the differential mechanisms of WMJ-J-09’ actions in regulating these two signaling pathways remain to be delineated. It is likely that these two signaling cascades converge in survivin reduction and subsequent cell death. Additional work is needed to further characterize whether WMJ-J-09’s growth-inhibition and apoptotic effects involve SHP-1 or other signaling molecules.

In keeping with previous studies that mithramycin A, a Sp1 inhibitor, markedly reduced basal survivin levels in colorectal cancer (Hsu et al., 2012) and breast cancer (Chuang et al., 2017) cells, we noted that mithramycin A also caused survivin reduction in FaDu cells (unpublished data). WMJ-J-09, however, significantly increased Sp1 transcriptional activity in FaDu cells (unpublished data). It suggests that WMJ-J-09’s inhibitory effect on survivin expression does not involve Sp1. The underlying mechanisms by which WMJ-J-09 induces Sp1 activation, and whether this activation leads to p21^*cip/Waf*^ induction in FaDu cells, as suggested in another study (Gartel et al., 2000), remain to be investigated. It was previously reported that acetylation status of Sp1 modulates its ability to transactivate target genes (Swingler et al., 2010; Waby et al., 2010). WMJ-J-09 appears to inhibit HDACs to increase acetylation of cellular signaling molecules like Sp1 in addition to histone 3 and α-tubulin as shown in this study. Eighteen HDACs are known in humans that are divided into four classes based on homology. Class I consists of HDAC 1, 2, 3, and 8. Class II HDACs are further grouped into two subgroups, Class IIA (HDAC 4, 5, 7, and 9) and Class IIB (HDAC 6 and 10). Class III consists of sirtuins (SIRT1-7) and HDAC 11 is the only member of Class IV (Bolden et al., 2006). Aberrant activation of HDACs, most notably Class I and Class II HDACs, have been implicated in tumor progression in most cancers (West and Johnstone, 2014). In this study, we noted that HDAC6-flag (a Class II HDAC) or HDAC8-flag (a Class I HDAC) significantly reduced WMJ-J-09-induced α-tubulin acetylation. Together these findings suggest that alteration of cellular acetylation status contributes to WMJ-J-09-induced FaDu HNSCC cell death. Further investigations are needed to explore whether WMJ-J-09’s anti-tumor mechanisms involves other HDAC isoforms besides HDAC6 and HDAC8.

The limitations of this study are as follows: Due to the lack of the *in vivo* pharmacokinetic models in our lab, we have not performed the pharmacokinetic experiments showing WMJ-J-09’s serum concentration, intrinsic clearance and half-life after administration. In addition, Quesnelle et al. (2012) recently showed that HNSCC cell line-derived xenografts, unlike HNSCC patients, respond to EGFR inhibition. There is clearly a need for the development of preclinical models in order to reliably assess clinical activity of novel compounds. Compared to xenografts derived from immortalized cell lines, patient-derived tumor xenograft (PDX) is emerging as a model that may better reflect human cancer behavior (Siolas and Hannon, 2013; Lodhia et al., 2015). To overcome these limitations, we will collaborate with other investigators and continue to explore WMJ-J-09’s pharmacokinetic properties after clarification of its pharmacological mechanisms. Additional work will also be required to establish PDX model to confirm WMJ-J-09’s anti-HNSCC activities.

## Conclusion

We demonstrated in this study that WMJ-J-09 exhibits anti-tumor effects via LKB1-AMPK-p38MAPK-p63-survivin and/or p21^*cip/Waf*^ cascade in FaDu HNSCC cells. WMJ-J-09’s actions in inducing cell death may also involve HDACs inhibition and tubulin assembly disruption. The precise mechanisms underlying these activities remain to be fully characterized. Together these observations suggest that WMJ-J-09 is a potential lead compound in the development of anti-tumor agents.

## Author Contributions

C-SY, C-SC, W-JH, Y-FH, and M-JH designed the experiments. S-WH, P-YL, M-CY, CS, and M-JH performed the experiments. C-SY, C-SC, W-JH, S-WH, Y-FH, and M-JH analyzed the data. W-JH contributed reagents/synthesized WMJ-J compounds. Y-FH and M-JH wrote the paper.

## Conflict of Interest Statement

The authors declare that the research was conducted in the absence of any commercial or financial relationships that could be construed as a potential conflict of interest.

## Abbreviations

AMPK: AMP-activated protein kinase
FBS: fetal bovine serum
HAT: histone acetyltransferase
HDAC: histone deacetylase
HNSCC: head and neck squamous cell carcinoma
HRP: horseradish peroxidase
LKB1: liver kinase B1
MTT: 3-[4, 5-dimethylthiazol-2-yl]-2, 5-diphenyltetrazolium bromide
PI: propidium iodide
