# Supplementary material for: A Novel Hydroxamate-Based Compound WMJ-J-09 Causes Head and Neck Squamous Cell Carcinoma Cell Death via LKB1-AMPK-p38MAPK-p63-Survivin Cascade
Source: Front Pharmacol. 2018 Mar 1;9:167. doi: 10.3389/fphar.2018.00167 (PMC5837967; doi:10.3389/fphar.2018.00167)
Supplement: Supplementary file 1 [file Data_Sheet_1.PDF]

Supplement Information

Supplement Methods

Synthesis of WMJ-J compounds:

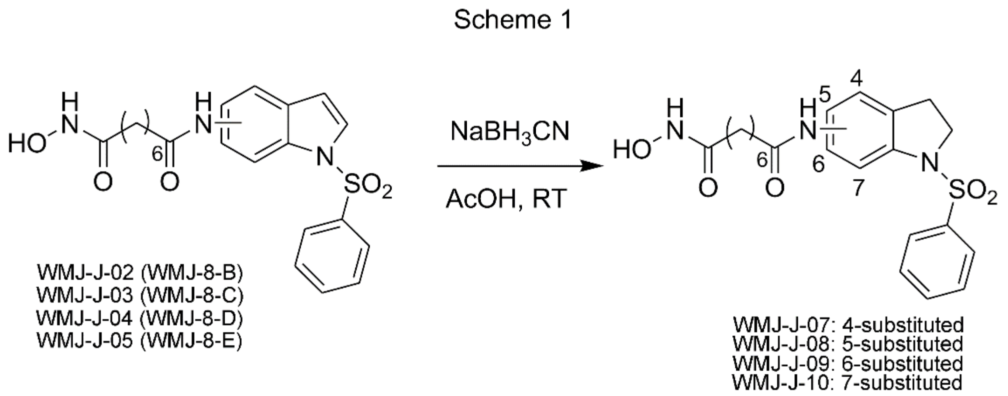

General procedure for the preparation of compounds **WMJ-J-07~10**.
WMJ-J-01 (WMJ-8-A) and **WMJ-J-06 (WMJ-8-F)** were synthesized as described previously (Chuang et al., 2017). To a solution of compounds **WMJ-02~05**(Chuang et al., 2017) (200 mg, 0.42 mmol) in AcOH (10 mL) was added $NaBH_3CN$ (53 mg, 0.84 mmol). The resulting solution was stirred at RT for 2 h. The reaction mixture was neutralized with 37% NH4OH, extracted with CH_2Cl_2 (50 mL x 3). The combined organic layer was dried over Na_2SO_4, filtered and the solvent removed in vacuum. The residue was purified by SiO_2 (1% MeOH/CH_2Cl_2) to give desired compounds.

***N*-Hydroxy 4-(1-benzenesulfonyl-indoline-4-ylcarbamoyl) heptanamide (WMJ-J-07)**
^{1}H-NMR (300MHz, DMSO-d_6) δ: 10.32 (s, 1H), 9.80 (s, 1H), 8.66 (s, 1H), 7.76 (m, 2H), 7.65 (t, *J* = 6.2 Hz, 1H), 7.55 (m, 2H), 7.41 (m, 1H), 7.35 (m, 2H), 3.88 (t, *J* = 8.2 Hz, 2H), 2.80 (t, *J* = 8.3 Hz, 2H), 2.23 (t, *J* = 7.3 Hz, 2H), 1.92 (t, *J* = 7.3 Hz, 2H), 1.47 (m, 4H), 1.23 (m ,4H); ^{13}C-NMR (125 MHz, DMSO-d_6) δ: 171.0, 169.1, 141.9, 136.1, 135.3, 133.8, 129.5, 127.9, 127.1, 124.3, 118.1, 110.1, 59.8, 49.9, 35.7, 32.2, 28.3, 28.3, 25.9, 25.0, 24.9.

***N*-Hydroxy 5-(1-benzenesulfonyl-indoline-4-ylcarbamoyl) heptanamide (WMJ-J-08)**
^{1}H-NMR (300MHz, DMSO-d_6) δ: 10.32 (s, 1H), 9.65 (s, 1H), 8.64 (s, 1H), 7.87 (s, 1H), 7.46 (d, *J* = 3.0 Hz, 1H), 7.41 (d, *J* = 8.8 Hz, 1H), 7.20 (dd, *J* = 1.8, 8.8 Hz, 1H),

6.57 (s, 2H), 6.40 (d, *J* = 2.9 Hz, 1H), 5.26 (s, 2H), 3.67 (s, 6H), 3.59 (s, 3H), 2.26 (t, *J* = 7.3 Hz, 2H), 1.93 (t, *J* = 7.2 Hz, 2H), 1.45-1.60 (m, 4H), 1.25-1.30 (m ,4H); ^{13}C-NMR (125 MHz, DMSO-d_6) δ: 171.0, 169.1, 136.4, 136.0, 135.8, 133.7, 132.8, 129.4, 127.1, 118.2, 116.4, 114.7, 50.2, 36.3, 32.2, 28.4, 27.5, 25.0.

***N*-Hydroxy 6-(1-benzenesulfonyl-indoline-4-ylcarbamoyl) heptanamide (WMJ-J-09)**

^{1}H-NMR (300MHz, DMSO-d_6) δ: 10.34 (s, 1H), 9.92 (s, 1H), 8.67 (s, 1H), 7.84 (m, 3H), 7.65 (t, *J* = 6.7 Hz, 1H), 7.57 (t, *J* = 7.8 Hz, 2H), 7.25 (dd, *J* = 1.8, 8.1 Hz, 1H), 7.04 (d, *J* = 8.1 Hz, 1H), 3.88 (d, *J* = 8.3 Hz, 2H), 2.82 (t, *J* = 8.3 Hz, 2H), 2.29 (t, *J* = 7.5 Hz, 2H), 1.95 (t, *J* = 7.5 Hz, 2H), 1.28 (m, 4H), 1.17 (m ,4H); ^{13}C-NMR (125 MHz, DMSO-d_6) δ: 171.2, 169.1, 141.5, 138.8, 136.0, 133.8, 129.4, 127.2, 126.1, 125.2, 114.5, 105.6, 50.4, 36.4, 32.3, 28.4, 26.7, 25.1, 25.0.

***N*-Hydroxy 7-(1-benzenesulfonyl-indoline-4-ylcarbamoyl) heptanamide (WMJ-J-10)**

^{1}H-NMR (300MHz, DMSO-d_6) δ: 10.33 (s, 1H), 9.36 (s, 1H), 8.66 (s, 1H), 7.84 (d, *J* = 8.1 Hz, 1H), 7.69 (t, *J* = 7.1 Hz, 1H), 7.51 (m, 4H), 7.11 (t, *J* = 7.7, 1H), 6.85 (d, *J* = 7.1 Hz, 1H), 4.02 (t, *J* = 7.5 Hz, 2H), 2.36 (t, *J* = 7.5 Hz, 2H), 1.96 (m, 2H), 1.56 (m, 4H), 1.30 (m ,4H); ^{13}C-NMR (125 MHz, DMSO-d_6) δ: 171.0, 169.1, 138.4, 136.0, 134.1, 131.8, 129.3, 127.3, 121.7, 120.4, 53.1, 36.6, 32.2, 28.4, 25.0, 24.8.

Supplement Figures

Supplement Fig. S1

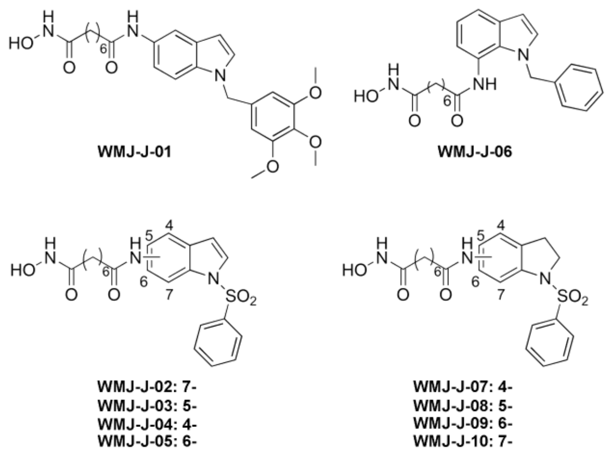

Supplement Fig. S2

A

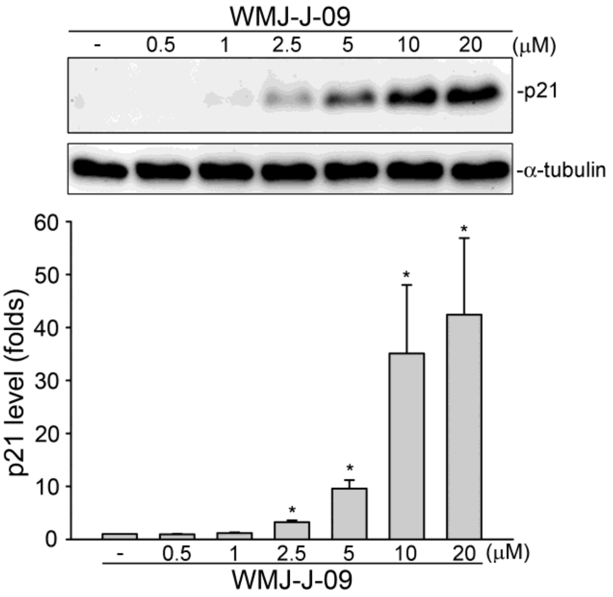

B

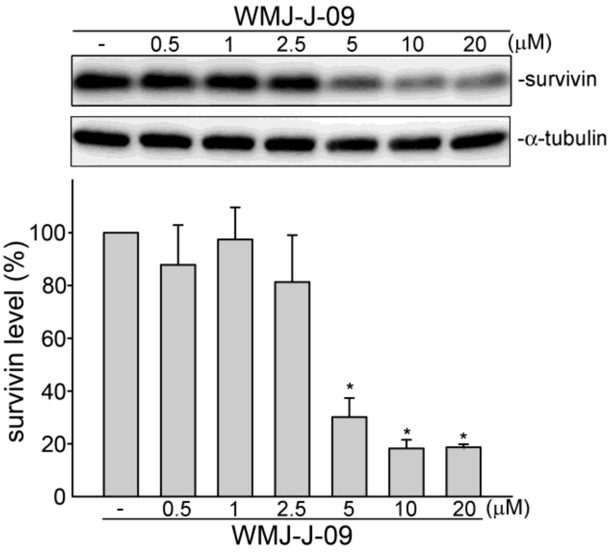

Supplement Fig. S3

A

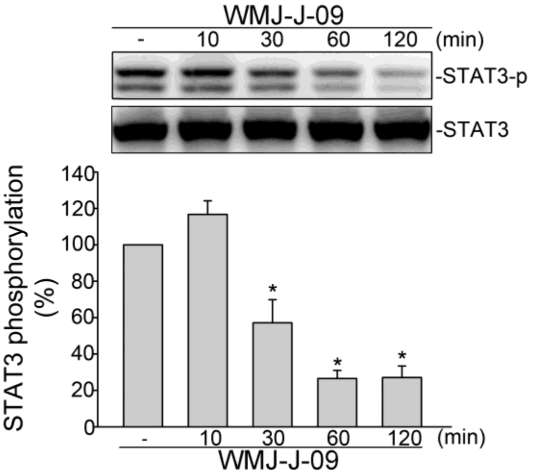

B

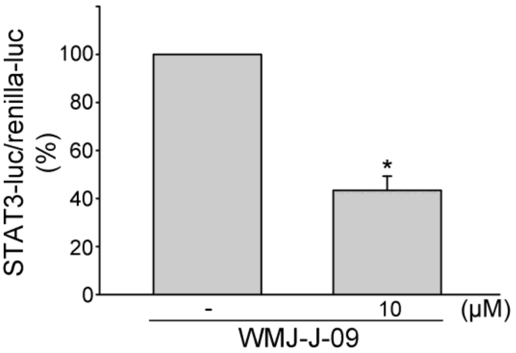

C

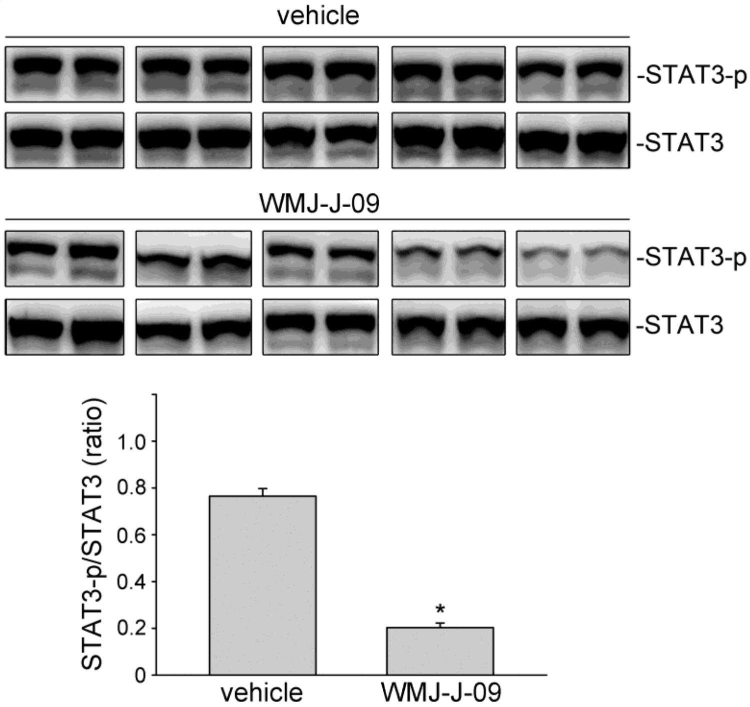

Supplement Figure legends

Supplement Figure S1. Chemical structures of WMJ-J compounds.

Supplement Figure S2. Effects of WMJ-J-09 on p21 and surviving levels in SCC25 cells.

SCC25 cells were treated with vehicle or WMJ-J-09 at indicated concentrations for 24 h. Protein levels of $p21^{cip/Waf}$ (A) and survivin (B) were determined by immunoblotting. Each column represents the mean ± S.E.M. of six independent experiments (Statistically significant differences were determined using the Kruskal-Wallis test. *$p < 0.05$, compared with the control group).

Supplement Figure S3. WMJ-J-09 caused STAT3 dephosphorylation and inactivation in

(A) Cells were treated with vehicle or WMJ-J-09 at 10 μM for indicated periods. The extent of STAT3 Tyr705 phosphorylation was determined by immunoblotting. Each column represents the mean ± S.E.M. of six independent experiments (Statistically significant differences were determined using the Kruskal-Wallis test. *$p < 0.05$, compared with the control group). (B) Cells were transiently transfected with STAT3-luc reporter construct and renilla-luc for 24 h followed by the treatment with WMJ-J-09 at 10 μM for another 24 h. Reporter assay was performed as described in the *Materials and methods* section. Each column represents the mean ± S.E.M. of five independent experiments performed in duplicate (Statistically significant differences were determined using the Mann-Whitney test. *$p < 0.05$, compared with the control group). (C) Nude mice bearing xenografts of FaDu cells were treated intraperitoneally with WMJ-J-09 20 mg/kg/day for 23 days. The control group received vehicle only. After 23 days of treatment, mice were sacrificed and protein lysates obtained from five randomly selected xenograft tumors were subjected to immunoblotting for assessing STAT3 or phosphorylated STAT3 levels. Values represents the mean ± S.E.M of five tumors from each group performed in duplicate (Statistically significant differences were determined using the Student's t-test. $^{*}p < 0.05$ as compared with the vehicle-treated control group). Technical replicates were used to ensure the reliability of single values for each experiment.

References:

Chuang YF, Huang SW, Hsu YF, Yu MC, Ou G, Huang WJ, *et al.* (2017). WMJ-8-B, a novel hydroxamate derivative, induces MDA-MB-231 breast cancer cell death via the SHP-1-STAT3-survivin cascade. Br J Pharmacol 174**:** 2941-2961.
